# Influence of radiation exposure to delayed fetal growth in wild Japanese monkeys after the Fukushima accident

**DOI:** 10.3389/fvets.2023.1151361

**Published:** 2023-04-26

**Authors:** Shin-ichi Hayama, Setsuko Nakanishi, Aki Tanaka, Fumiharu Konno, Yoshi Kawamoto, Toshinori Omi

**Affiliations:** ^1^School of Veterinary Medicine, Nippon Veterinary and Life Science University, Musashino, Tokyo, Japan; ^2^Tohoku Wildlife Management Center, Sendai, Miyagi, Japan; ^3^School of Veterinary Nursing and Technology, Nippon Veterinary and Life Science University, Musashino, Tokyo, Japan

**Keywords:** gestational development, fetal body weight, fetal head size, nuclear accident, radiation exposure, Japanese monkeys, teratology, birth defects

## Abstract

Wild Japanese monkeys (*Macaca fuscata*) were exposed to radiation after the Fukushima Daiichi nuclear accident in 2011. To clarify the biological effects of radiation exposure on their fetal growth, pregnant monkeys and their fetuses were analyzed. These animals were collected between 2008 and 2020 (before and after the accident in 2011) living in Fukushima City, approximately 70 km from the nuclear power plant. Multiple regression analyses were conducted with fetal body weight (FBW) and fetal head circumference (FHS) as objective variables, and maternal and fetal factors as explanatory variables. The maternal factors were relative exposure dose rate (REDR), age, body weight, body length, fat index, and parity. The fetal factors were crown ramp length (CRL) and sex. Multiple regression analyses showed that FBR and FHS growth were positively associated with CRL, maternal body length, and negatively associated with REDR. Since the relative growth of FBR and FHS to CRL decreased with increasing REDR, radiation exposure due to the nuclear accident may have contributed to the delayed fetal growth observed in Japanese monkeys.

## 1. Introduction

**Figure 1:**
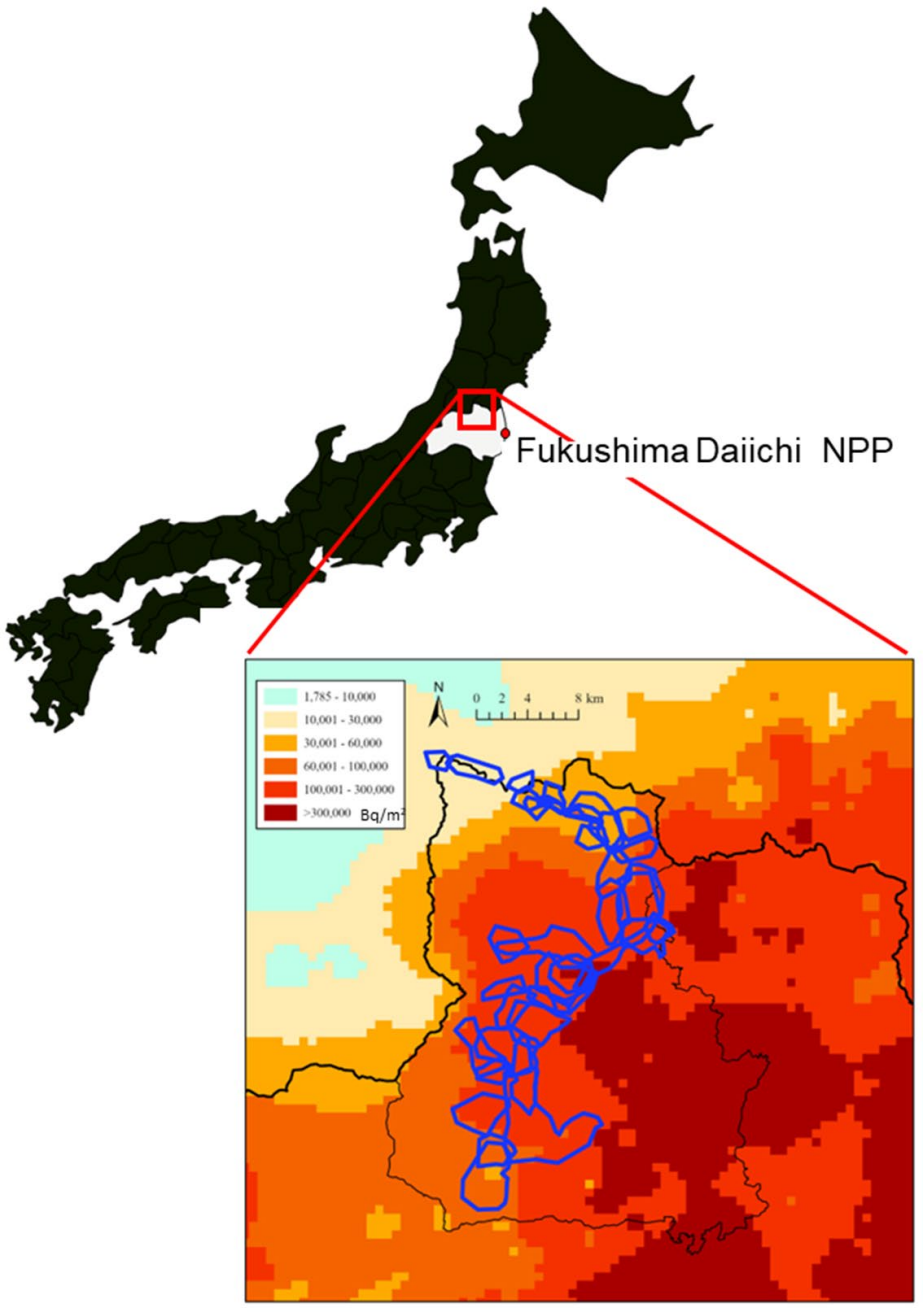
Soil contamination levels by radiocesium concentrations (Bq/m^2^) and the distribution of monkey troops (irregular enclosed blue outlines) in Fukushima City. This map was generated according to the soil contamination map created by the Ministry of Education, Culture, Sports, Science and Technology (converted to values for July 2, 2011).

The accident at the Fukushima Daiichi Nuclear Power Plant (FDNPP) in March 2011 exposed many people and wildlife to radioactive materials. In order to mitigate the damage to crops, the population of Japanese monkeys in Fukushima City has been systematically managed since 2008 in accordance with the laws and regulations established by Fukushima Prefecture. Several papers have been published on the health effects of radiation exposure on Japanese monkeys in Fukushima City (1, 2), located approximately 70 km from the FDNPP (Figure 1). As a result of this accident, this population of Japanese monkeys (*Macaca fuscata*) became the first wild primate in the world to be exposed to radiation (2).

The effects of long-term exposure to low doses of radiation on the fetus are among the many health concerns. Low birth weight and high rates of microcephaly have been reported in children born to mothers exposed to the atomic bombs in Hiroshima and Nagasaki (3). Another paper (4) has shown that these children have intellectual disability due to abnormal brain development. In wild animals, Møller et al. (5) reported that the brain weights of birds captured near the Chernobyl NPP were lower than those of birds captured elsewhere. Our study also found that the body weight and head size relative to the crown–rump length (CRL) were significantly lower in fetuses conceived after the FDNPP accident (2011–2016) than in fetuses conceived prior to the FDNPP accident (2008–2010) (2). No other studies from Chernobyl or Fukushima have tracked fetal development over time in the same wildlife populations or compared fetal development before and after long-term radiation exposure (2). However, this previous study did not account for the fetal radiation exposure dose-rate.

In cows, the accumulation of radioactive materials is higher in the fetus than in the mother (6). However, in Japanese monkeys, the accumulation of radioactive materials in the fetus is unknown, it is difficult to estimate the exact exposure dose-rate. In this study, the internal dose rate in the mother was adopted as an indicator of the relative dose rate to the fetus.

In some primates, including humans, fetus growth is known to be affected by maternal age, parity, body size and nutritional status, and the sex of the fetus (7–10). The purpose of this study is to determine the factors that influence the growth of fetal body weight (FBW) and fetal head size (FHS) relative to CRL. In this study, the maternal factors (body weight, body length, parity, fat index, age and the relative exposure dose-rate) and the fetal factors (CRL and sex) were evaluated by multiple regression analysis. Because monkeys are taxonomically close to humans, this study may be useful for research on the health effects of radiation exposure on humans.

## 2. Materials and methods

### 2.1. Animals and ethics

This study was approved by the Institutional Animal Care and Use Committee of Nippon Veterinary and Life Science University (No. 2022S-1). All experiments were performed in accordance with relevant guidelines and regulations. Carcasses of Japanese monkeys were provided by Fukushima City. Monkeys were culled to prevent crop damage with the permission of the governor of Fukushima Prefecture, according to the Fukushima Japanese Monkey Management Plan, which was established based on the Wildlife Protection and Hunting Management Law. Monkeys were captured using box traps and euthanized by a gun by licensed hunters at the request of Fukushima City. The methods for capture and euthanasia were in accordance with the guidelines of the management plan and do not present an ethical concern. This euthanasia method was also in accordance with guidelines published by the Wildlife Research Center of Kyoto University (11). The Japanese monkeys inhabiting the study area were not listed as an endangered species on the Japanese Red List, as revised by the Ministry of the Environment in 2012 (12). The sample size of monkey mothers and fetuses was 32 before and 54 after the accident.

### 2.2. Mothers and muscle samples

Pregnant Japanese monkey carcasses were collected from 2008 to 2020, transported under refrigerated conditions to our laboratory, and subjected to necropsies. The body weight (BW) of each monkey was measured in grams. Body length (BL) was measured in millimeters as the straight-line distance from the top of head to the rump, at the dorsal surface of the sciatic protuberances while the monkey was in a recumbent position. During the necropsy, fat indices (FI) to evaluate the nutritional status were calculated that the ratio of the mesenteric fat weight to body weight was proportional to the percentage of body fat in Japanese monkeys (2). The FI was defined as mesenteric fat weight (g) divided by body weight (g) and multiplied by 1,000. Age was classified as sub-adult or adult by tooth eruption; in adults (13), all permanent teeth are erupted, corresponding to an age of at least 7 years old. Parity was assessed as primiparous or multiparous. Nipples of primiparous animals were not developed because they were not sucked by the young at the time of capture (14). Multiparous individuals had elongated teats on one or both sides.

During the necropsy after the FDNPP accident, 500–1,000 g of muscle tissue from the hind limb of each mother was collected; this tissue type was used because organs weighing 500 g or more were required to measure the radiocesium concentration. The muscle tissue was stored at −30°C until it was used for radioactivity measurements.

### 2.3. Fetuses

After the fetuses were removed from the uterus during the necropsy, FBW was measured in grams, and CRL (i.e., the length of the fetus from the top of its head to bottom of torso) was measured in millimeters. CRL is a common somatometric measure for age assessment in physical and neurological examinations (15).

Fetuses were preserved in 10% neutral buffered formalin. FHS was defined as the product of the biparietal diameter and occipital frontal diameter. The biparietal diameter is a basic biometric parameter used to assess fetal size and is the maximum width of the head. The occipital frontal diameter was measured as the maximum length between the forehead and occipital region.

All specimens were measured by the same person in millimeters, using a caliper. Specimens with a CRL greater than 80 mm (fetal age of about 3 months or greater) were included in the analysis. This is because in fetuses of this size, the cranium is ossified and there is less error in the external measurements.

The fetuses were divided into those from pregnancies between 2008 and 2010 (pre-accident) and those from pregnancies in 2011 or later (post-accident). The average conception date of Japanese monkeys in Fukushima City is November 19 (SD = 29.2 days) (16), and the average gestation period is 180 days (17). Therefore, the fetuses conceived during the mating season in 2010, the year before the accident, were likely exposed to radiation from the accident in March 2011 for fewer than about 60 days in the second trimester of pregnancy. Because the sensitivity to radiation exposure in the fetal period is highest in the first trimester of pregnancy (4, 18, 19), fetuses conceived in 2010 were included in the pre-accident group in this study.

### 2.4. Radioactivity measurements

The muscle radiocesium concentration was measured in mothers that were pregnant after the accident. The concentration of radioactive cesium in the muscle tissue before the accident was treated as 0 Bq/kg (muscle samples were not collected for this group).

The radioactivity of radiocesium in the muscle samples was analyzed using a germanium semiconductor spectrometer (GC2020-7500SL-2002 CSL; Canberra, Meriden, CT) and a NaI (T1) scintillation detector (AT1320A; Atometex, Minsk, Belarus). Data were corrected to the background radiation dose in the measurement environment as-needed. ^134^Cs was detected using 604.70 and 795.85 keV gamma-rays, whereas ^137^Cs was detected using 661.6 keV gamma-rays. The radioactivity of radiocesium was adjusted to the value on the day of capture based on its physical half-life. The limit of detection was 10 Bq/kg. The muscle radiocesium concentration was calculated as the combined concentration of ^134^Cs and ^137^Cs per kilogram of fresh muscle.

### 2.5. Relative exposure dose-rate

Urushihara et al. (20) estimated the radiocesium dose-rate using the ERICA tool [version 1.2; (21)], with some modifications, and determined dose conversion coefficients (DCCs) using the equation provided by the ERICA tool for Japanese monkeys [Supplementary Table S4 in Urushihara et al. (20)]. The total dose-rate in the mother should include external and internal dose-rate, the total dose-rate and internal dose-rate are highly correlated in Japanese monkeys in Fukushima [r = 0.98, *p* < 0.0001; Urushihara et al. (20)]. Then, the estimated internal exposure dose-rate in the mother was used as an indicator of the relative exposure dose-rate (REDR) for the fetus by estimating the muscle radiocesium concentration in the mother using the DCCs obtained by Urushihara et al. (20).

### 2.6. Statistics

The Shapiro–Wilk test was used to test the normality of continuous variables. Wilcoxon single rank test was performed to compare medians for mother monkey body weight, body length, and fat index, and fetal CRL, body weight and head size by pre- and post-accident. Multiple non-parametric linear regressions were performed with dependent variables (FBW and FHS) and explanatory variables, including maternal factors (BW, BL, Parity, FI, and Age, and REDR) and fetal factors (CRL and Sex). Stata/IC 16 (StataCorp, College Station, TX) was used for all analyses. Two-sided tests were used with a 5% significance level.

## 3. Results

Descriptive statistics for Japanese monkey mothers and fetuses in Fukushima are shown in Table 1 with median (range) for mother monkey body weight (g), body length (mm), and fat index, and fetal CRL (mm), body weight (g) and head size (mm^2^) by pre- and post-accident with value of *p*s, respectively.

**Table 1:** Characteristics of mothers (*N* = 86) and fetuses (*N* = 86) based on Japanese monkey carcasses collected in Fukushima, Japan from 2008 and 2020.

Characteristics	Pre-accident (2008–2010)			Post-accident (2011–2020)			*p*-value
	Number of monkeys	Median	Range	Number of monkeys	Median	Range	
Mothers							
Pregnant Year	32			54			
Age (years)							
Sub-Adult	5			11			
Adult	27			43			
Parity							
Primipara	12			9			
Multipara	20			45			
Body weight (g)	32	9,967	7,115–13,685	54	9,892	7,310–12,560	0.47
Body length (mm)	25	563	517–616	54	551	453–627	0.32
Fat index	32	1.56	0.24–4.91	54	1.32	0.33–2.89	0.14
Relative exposure dose-rate (μGy/day)	32	0	0	54	1.16	0–13.6	
Fetus							
Sex							
Male	13			34			
Female	19			20			
CRL (mm)	32	136	84–191	54	136	83–200	0.33
Body weight (g)	32	225	39–623	54	202	39–676	0.78
Head size (mm^2^)	32	2,185	636–4,621	54	1981	702–4,690	0.75

**Figure 2:**
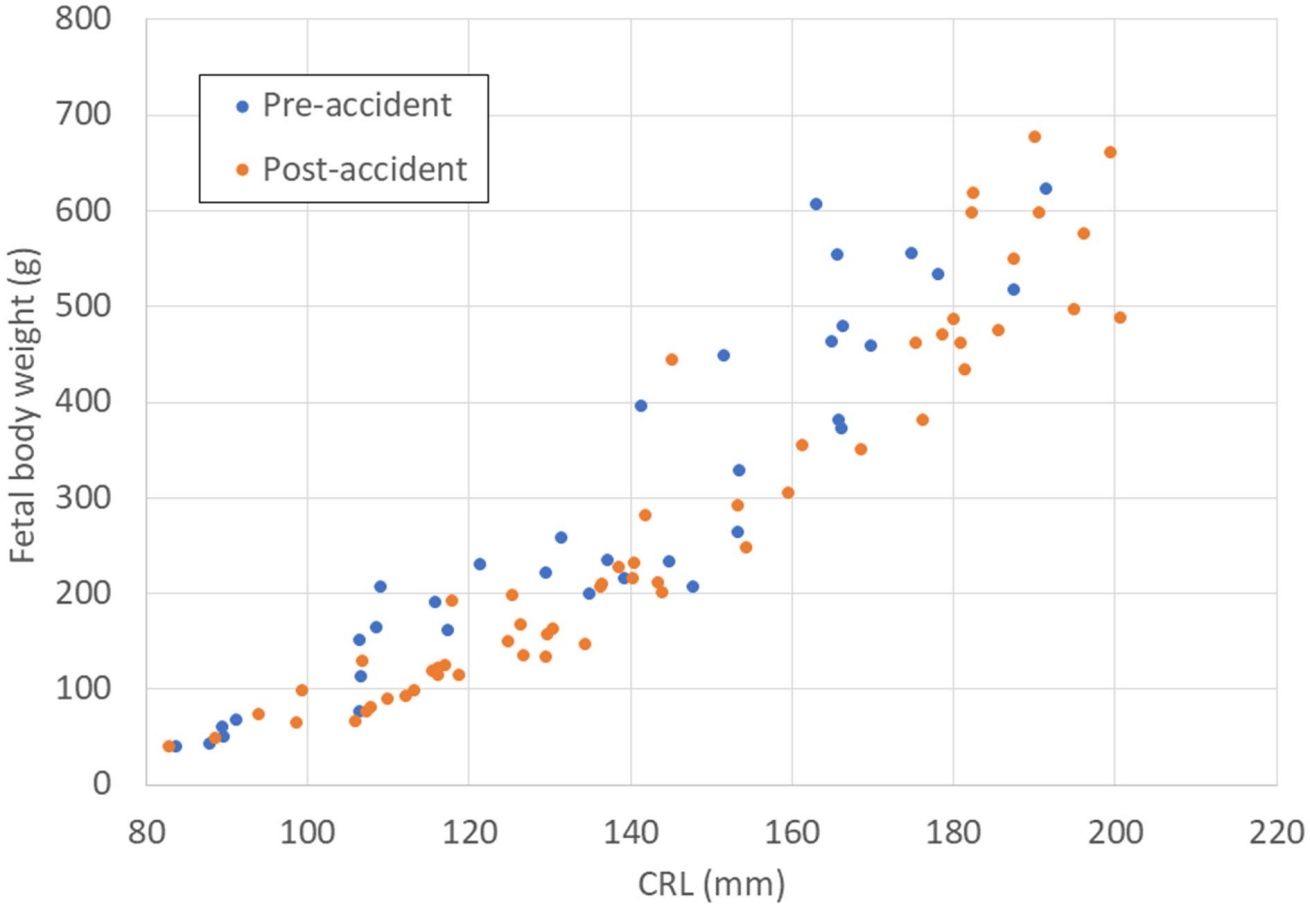
Relationship between CRL (mm) and FBW (g) in fetal Japanese monkeys before and after the Fukushima Daiichi nuclear power plant accident. Blue circles indicate fetal monkeys before the accident; red circles indicate fetal monkeys after the accident. CRL, crown–rump length; FBW, fetal body weight.

**Figure 3:**
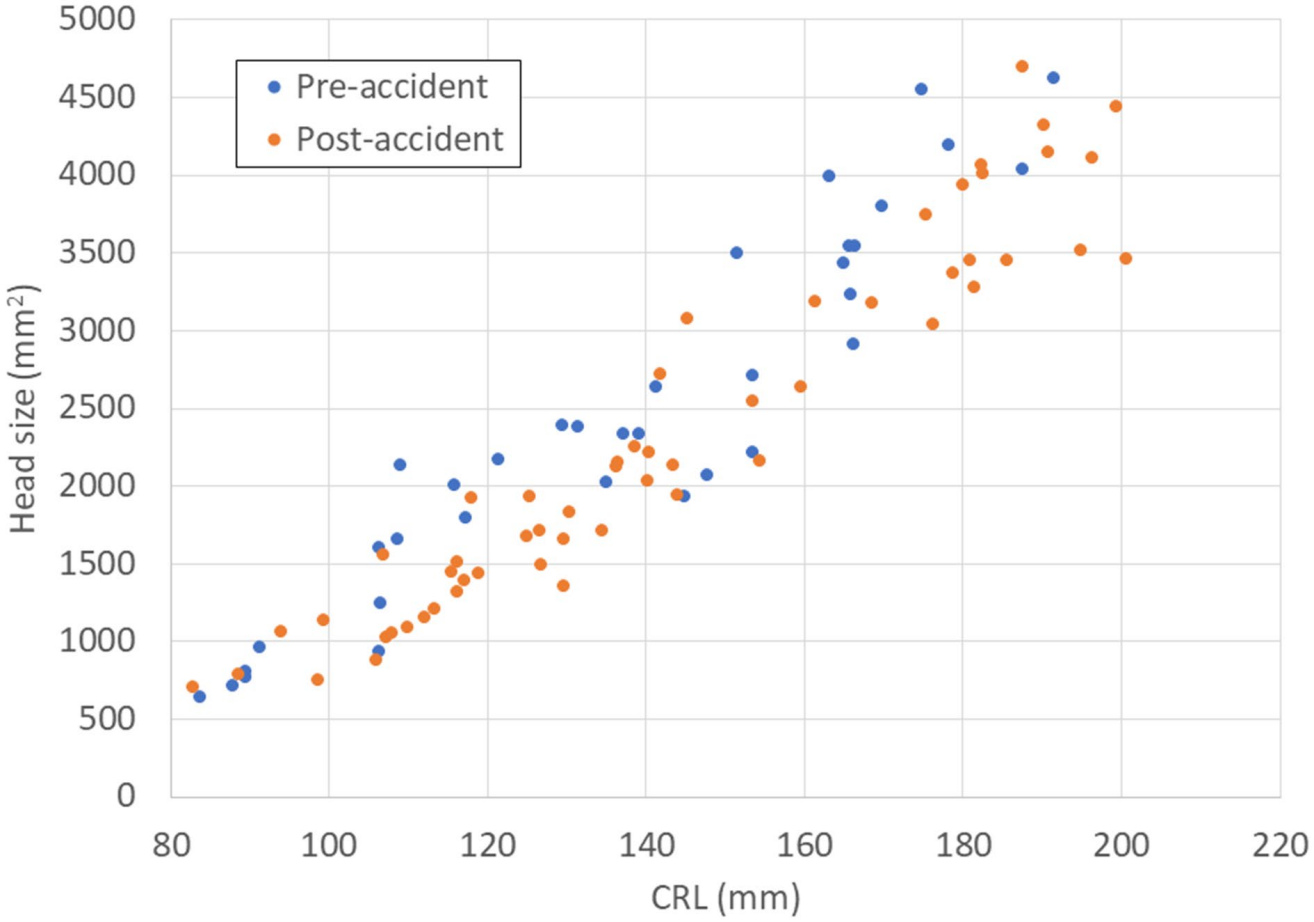
Relationship between CRL (mm) and FHS (mm^2^) in fetal Japanese monkeys before and after the Fukushima Daiichi nuclear power plant accident. Blue circles indicate fetal monkeys before the accident; red circles indicate fetal monkeys after the accident. CRL, crown–rump length; FHS, fetal head size.

The growth of FBW and FHS relative to CRL tended to be delayed after the accident. Associations between FBW and CRL in pre- and post-accident groups are described in Figure 2, and associations between FHS and CRL in pre- and post-accident groups are described in Figure 3.

Table 2 shows the results of multiple non-parametric linear regression analyses of fetal body weight and head size of Japanese monkeys collected in Fukushima from 2008 to 2020 as dependent variables and associated factors as independent variables including age, delivery, fat index, relative exposure dose-rate (μGy/day), body weight, body length, fetal CRL, and sex. Both fetal body weight and fetal head size were positively associated with CRL (*p* < 0.001) and mother body length (FBW: *p* < 0.02; FHS: *p* < 0.05), and negatively associated with relative exposure dose-rate (FBW: *p* < 0.01; FHS: *p* < 0.05).

**Table 2:** Multiple non-parametric linear regression analyses of dependent variables as fetal body weight and head size of Japanese monkeys collected in Fukushima from 2008 to 2020 and associated factors (*n* = 86).

Independent variables	Dependent variables									
	Fetal body weight					Fetal head size				
	Coefficient	Standard error	*Z*	95% Cl	*P* > |*Z*|	Coefficient	Standard error	*Z*	95% Cl	*P* > |*Z*|
Mother										
Age (Sub-adult vs. Adult)	−21.84	18.28	−1.19	(−57.66) – (−13.99)	0.23	−202.10	118.8	−1.7	(−434.96) – 30.74	0.09
Delivery (Primipara vs. Multipara)	5.56	17.3	0.32	(−28.36) – (−39.48)	0.75	134.11	126.05	1.06	(−112.93) – 381.16	0.29
Fat index	−2.09	15.75	−0.13	(−32.97) – 28.79	0.89	−42.15	82.64	−0.51	(−204.12) – 119.83	0.61
Relative exposure dose-rate (μGy/day)	−5.08	2.07	−2.45	(−9.15) – (−1.01)	**0.01**	−29.26	14.93	−1.96	(−58.51) – 0.01	**0.05**
Body weight (g)	0.009	0.006	1.54	(−0.003) – 0.21	0.12	0.06	0.04	1.7	(−0.01) - 0.13	0.09
Body length (mm)	0.52	0.23	2.26	0.07–0.97	**0.02**	2.93	2.93	1.47	0.40–5.82	**0.05**
Fetus										
CRL (mm)	5.18	0.21	24.85	4.77–5.59	**<0.001**	32.84	1.25	26.35	30.40–35.28	**<0.001**
Sex (Male vs. Female)	8.89	11.94	0.75	(−14.50) – 32.29	0.46	15.24	74.58	0.2	(−130.94) – 161.42	0.84

*p*-values less than 0.05 were considered to be statistifically significant and were shown in bold; Coefficient: values for the regression equation for predicting the dependent variable from the independent variable; CI: Confidence Interval.

No significant relationships were found with fetal growth parameters and other factors.

## 4. Discussion

In this study, the relationship between fetal growth (FBW and FHS) and various factors was analyzed in wild Japanese monkeys exposed to radiation during the FDNPP accident. The only factor that was negatively associated with relative growth parameters (both FBW and FHS) was REDR.

This is the first study to clarify the influence of radiation to delayed fetal growth in wild animals. Based on our results, the relative growth of fetuses is expected to return to the pre-exposure state if radiation exposure is reduced in the future. However, the radioactive cesium concentration in the muscle tissues in this population decreased year by year after the accident until 2015, with no additional decrease after 2016 (22). Therefore, it is not possible to predict when the growth rate will return to pre-accident levels.

Scherb and Hayashi (23) analyzed prefecture-specific spatiotemporal trends in the frequency of low birth weight in Japan using annual counts for 26.158 million live births from 1995 to 2018 provided by the Japanese Ministry of Health, Labor and Welfare. A logistic regression of low birth weight proportions against the additional dose-rate after accidents adjusted for prefecture-specific spatiotemporal base-line trends yielded an odds ratio per μSv/h of 1.098 (95% confidence interval, 1.058–1.139, *p* < 0.0001). These results are not directly comparable to ours, but suggest similar conclusions.

CRL and BL were significantly associated with relative growth in FBW and FHS. Because CRL increases with fetal growth, a positive association is an inevitable consequence. BL also positively affects fetal growth as in human case studies (10). There are also reports that maternal height is associated with increased birth weight (9, 24). However, REDR, used the internal dose-rate in the mother,increases in proportion to the BL (20). The contribution of the muscle radiocesium concentration to the estimation of REDR may offset the effect of BL.

Maternal age, parity, and fetal sex were not significantly related to fetal growth. On the other hand, it has been reported that these factors are associated with increased birth weight in humans and captive macaques (7, 9). These factors have also been associated with fetal growth retardation (25). Hopper et al. (8) reported that maternal birth weight of rhesus monkeys did not correlate with infant birth weight, and increased parity of the mother was associated with higher birth weight of the infant.

In this study, it is possible that the exact age of the mothers and their history of parity were unknown, and thus a clear causal relationship with fetal growth could not be determined. Although there have been several reports of maternal pre-pregnancy weight and weight gain during pregnancy affecting birth weight (24, 26), it was not possible to obtain these data in this study, so a similar test could not be performed.

Jhonsenn et al. (10) used ultrasound to estimate fetal body weight from fetus measurements and examined the association between intrauterine growth of the fetus and maternal factors in humans. They found that male fetuses were heavier than female fetuses at the same gestational week and that maternal height and age were associated with increased fetal weight.

On the other hand, in this study (10), maternal weight and body mass index had no effect on fetal weight growth. In our study, FI and BW were not significantly related to fetal growth. This may be due to the large individual differences in maternal weight and nutritional status during pregnancy in both humans and monkeys. Japanese monkeys are conceived from the autumn to early winter and deliver in the spring, and body fat and weight fluctuate substantially during these periods. Monkeys eat large amounts of acorns and other foods in the fall and gain 20–30% body weight through early winter, followed by weight loss until minimum in the spring (27). This seasonal variation may weaken the relationship between FI or BW and fetal growth in Japanese monkeys.

## 5. Conclusion

This study evaluated maternal factors (weight, length, parity, fat index, age, and relative dose rate) and fetal factors (CRL and sex) by multiple regression analysis to identify factors associated with fetal body weight (FBW) and fetal head size (FHS) growth relative to CRL. The results revealed that only REDR had a significant negative association with the relative growth of FBW and FHS. However, REDR is a relative measure of convenience in this study. In addition, the actual exposure dose should be higher than the REDR because it is probably the minimum exposure for the fetus. The exact fetal exposure dose-rate needs to be clarified in future studies.

## Data availability statement

The raw data supporting the conclusions of this article will be made available by the authors, without undue reservation.

## Ethics statement

Carcasses of animals were provided by Fukushima City with the permission of the governor of Fukushima Prefecture under the Wildlife Protection and Hunting Management Law. The methods for capture and euthanasia were in accordance with the guidelines of the management plan by the Environmental Agency of Japanese government. This research was approved by the Animal Research Committee in Nippon Veterinary and Life Science University (#2022S-1).

## Author contributions

SN and FK collected specimens. S-iH, YK, and TO designed the study. AT analyzed the data. S-iH wrote the paper. All authors contributed to the article and approved the submitted version.

## Funding

This research was funded by the Cooperative Research Program of The Primate Research Institute (Kyoto University), Act Beyond Trust, The Promotion and Mutual Aid Corporation for Private Schools of Japan, The Sumitomo Foundation, and the Japan Society for the Promotion of Science (KAKENHI Grant nos. 25517008, 16K08087, and 20K06400).

## Conflict of interest

The authors declare that the research was conducted in the absence of any commercial or financial relationships that could be construed as a potential conflict of interest.

## Publisher’s note

All claims expressed in this article are solely those of the authors and do not necessarily represent those of their affiliated organizations, or those of the publisher, the editors and the reviewers. Any product that may be evaluated in this article, or claim that may be made by its manufacturer, is not guaranteed or endorsed by the publisher.
